# Hyperuricemia and Incident Cardiovascular Disease and Noncardiac Vascular Events in Patients with Rheumatoid Arthritis

**DOI:** 10.1155/2014/523897

**Published:** 2014-08-17

**Authors:** Daniel Kuo, Cynthia S. Crowson, Sherine E. Gabriel, Eric L. Matteson

**Affiliations:** ^1^Department of Medicine, Mayo Clinic College of Medicine, 200 1st Street SW, Rochester, MN 55905, USA; ^2^Department of Health Sciences Research, Mayo Clinic College of Medicine, 200 1st Street SW, Rochester, MN 55905, USA; ^3^Division of Rheumatology, Mayo Clinic College of Medicine, 200 1st Street SW, Rochester, MN 55905, USA

## Abstract

*Objective*. To evaluate whether hyperuricemia is a risk factor for cardiovascular disease (CVD) in patients with rheumatoid arthritis (RA). *Methods*. A population-based inception cohort of patients diagnosed between 1980 and 2007 with adult-onset RA was assembled. A comparison cohort of age- and sex-matched subjects without RA (non-RA) was also assembled. All clinically obtained uric acid values were collected. CVD and noncardiac vascular events were recorded for each patient. Cox proportional hazards models were used to assess the impact of hyperuricemia on development of CVD, mortality, and noncardiac vascular disease. *Results*. In patients without RA, hyperuricemia was associated with heart failure (HR: 1.95; 95% CI: 1.13–3.39) and CVD (HR: 1.59; 95% CI: 0.99–2.55). In patients with RA, hyperuricemia was not significantly associated with CVD but was significantly associated with peripheral arterial events (HR: 2.52; 95% CI: 1.17–5.42). Hyperuricemia appeared to be more strongly associated with mortality among RA patients (HR: 1.96; 95% CI: 1.45–2.65) than among the non-RA subjects (HR: 1.57; 95% CI: 1.09–2.24). *Conclusion*. In patients with RA, hyperuricemia was a significant predictor of peripheral arterial events and mortality but not of CVD.

## 1. Introduction

Patients with rheumatoid arthritis (RA) are at increased risk to develop cardiovascular disease (CVD) [1, 2] and experience cardiovascular mortality [3, 4]. Although treatments for RA have improved over time, cardiovascular mortality has remained relatively unchanged [4] and is responsible for 50 percent of premature deaths in patients with RA [5, 6]. Traditional cardiovascular risk factors including smoking, hypertension, hyperlipidemia, obesity, and diabetes are important factors but do not adequately account for the excess burden of CVD in RA patients [7].

There is growing evidence that serum uric acid (SUA) may play a role in CVD in the general population [8, 9]. Indeed epidemiologic studies have found that hyperuricemia appears to be an independent risk factor for hypertension [10], heart failure (HF) [11], coronary artery disease [12], and stroke [13]. Experimental studies have also shown that uric acid is a functionally active molecule that can contribute to proatherogenic processes including inflammation, endothelial dysfunction, and oxidative stress [14].

The role of hyperuricemia in RA has not been well studied and only a few papers have addressed the subject [15, 16]. However, hyperuricemia is common in the general population with a prevalence of 6–8% among healthy adults and as many as 1 in 3 adults with uncontrolled hypertension and several CVD risk factors [17]. Given the excess burden of cardiovascular disease in patients with RA and the potential role of serum uric acid, the purpose of this study was to examine the effects of hyperuricemia on CVD specifically in patients with RA.

## 2. Patients and Methods

### 2.1. Study Population

An inception cohort of all cases of rheumatoid arthritis (RA) first diagnosed between January 1, 1980 and December 31, 2007 (*n* = 813) among Olmsted County residents ≥18 years of age was assembled as previously described [18]. The incidence date was defined as the earliest date at which the patient fulfilled at least 4 of the 7 American College of Rheumatology (ACR; formerly, the American Rheumatism Association) 1987 classification criteria for RA [19]. A comparison cohort of subjects without RA with similar age, sex, and calendar year was also assembled. All patients in both cohorts were followed up longitudinally through their entire medical records until death, migration from Olmsted County, or December 31, 2008. The study was approved by the Institutional Review Boards of the Mayo Clinic and Olmsted Medical Center.

### 2.2. Data Collection

All clinically obtained uric acid values were collected. Mayo Medical Laboratory reference ranges for adults by sex were used. Hyperuricemia was defined as serum uric acid (SUA) >8.0 mg/dL for males and >6.1 mg/dL for females.

Clinically available data for traditional cardiovascular risk factors were collected, including age, gender, race, smoking history, body mass index (BMI), diabetes mellitus, hypertension, and dyslipidemia. Data from the medical record were also collected for factors that could affect SUA levels including estimated glomerular filtration rate (GFR), alcoholism, low-dose aspirin, and urate lowering therapy.

CVD recorded for each patient included angina, myocardial infarction (MI; hospitalized or silent), revascularization procedures, and HF. Noncardiac vascular diseases (NCVD) included stroke, thromboembolism, and peripheral vascular disease [20]. Mortality data were also collected.

### 2.3. Statistical Methods

Descriptive statistics (means, percentages, etc.) were used to summarize the data. Poisson regression models with smoothing splines were used to examine trends in uric acid levels according to time since RA incidence/index date with adjustment for age and sex. The cumulative incidence of hyperuricemia adjusted for the competing risk of death was estimated [21]. These methods are similar to Kaplan-Meier method with censoring of patients who are still alive at last follow-up. However, patients who die before experiencing hyperuricemia are appropriately accounted for to avoid the overestimation of the rate of occurrence of hyperuricemia, which can occur if such subjects are simply censored.

Patients who were diagnosed with hyperuricemia prior to the diagnosis of RA, or prior to the index date for subjects in the non-RA comparison cohort, were excluded from the analysis of cumulative incidence. A sensitivity analysis was performed to examine the possibility that differences in testing rates might influence cumulative incidence results. A subset of uric acid tests was randomly selected from patients with RA to mimic the testing rate in patients without RA for the sensitivity analysis. Cumulative incidence comparisons between the cohorts were performed using methods by Gray [22].

Cox proportional hazards models were used to compare the rate of development of hyperuricemia between patients with RA and the non-RA comparison cohort. Cox models were also used to assess the impact of hyperuricemia on the development of CVD, NCVD, or mortality among patients with RA and non-RA subjects. Age was used as the time scale for these models to provide optimal adjustment for age under the assumption that age is likely the most important time determinate of cardiovascular disease. Subjects entered the model at the age they met criteria for RA and remained in the model until the age of each cardiac or vascular disease event. Subjects without events were censored at the age of death or last follow-up. The models were also stratified by sex. Traditional cardiovascular risk factors as well as factors that could affect both uric acid levels and the outcomes of interest (e.g., GFR, alcoholism, low-dose aspirin, and urate-lowering therapy) were included in these models as adjustors. Time-dependent covariates were used to model risk factors that developed over time. These time-dependent covariates allowed patients to be modeled as unexposed to the risk factor during the follow-up time prior to development of the risk factor and then change to exposed following development of the risk factor. Interactions between cohort and hyperuricemia were examined.

## 3. Results

The study population included 813 patients with RA and 813 subjects without RA. The average age at RA incidence (index date for the non-RA cohort) was 55.9 (SD 15.7) years and 556 (68%) were female in each cohort (Table 1). There was no difference in the presence of hyperuricemia at RA incidence/index date between cohorts (*P* = 0.22). Rates of testing of SUA were higher among patients with RA (0.77 tests per 1 person-year of follow-up) than among non-RA subjects (0.50 per 1 person-year of follow-up; *P* < 0.001).

**Figure 1:**
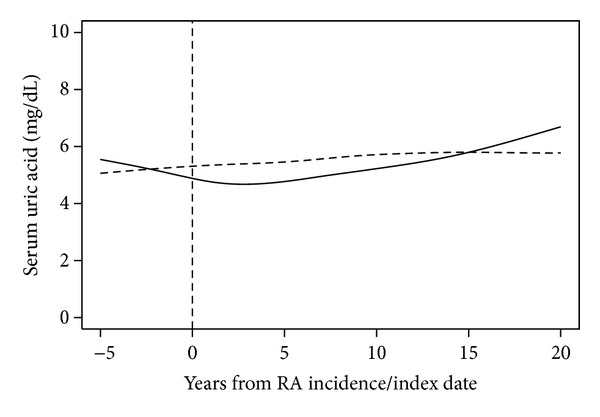
Trends in serum uric acid over time in patients with rheumatoid arthritis (RA; solid line) and without RA (dashed line).

There were no apparent assay changes in SUA ranges by calendar years of interest (figure not shown). Examination of trends in serum uric acid according to time since RA incidence/index date revealed that the average serum uric acid decreases in RA patients prior to diagnosis of RA (Figure 1). SUA remains lower in RA on average than in non-RA for the first 10 years of RA disease duration.

**Figure 2:**
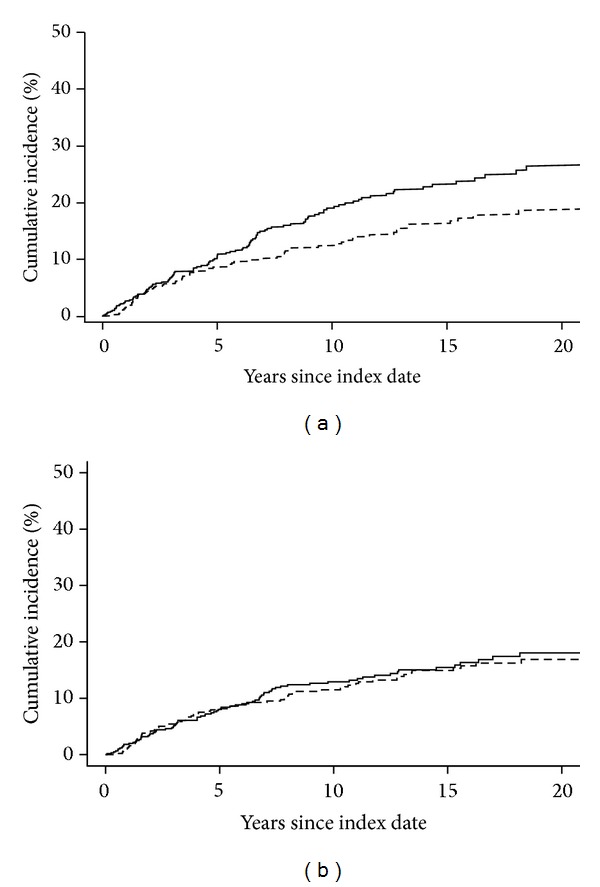
(a) Cumulative incidence of hyperuricemia in patients with rheumatoid arthritis (RA) versus subjects without RA (non-RA). The solid line is RA and dashed line is non-RA (*P* = 0.008). (b) Cumulative incidence of hyperuricemia in patients with RA (based on randomly selected uric acid tests using lower testing rate of the non-RA cohort) versus subjects without RA (non-RA). The solid line is RA and dashed line is non-RA (*P* = 0.64).

The cumulative incidence of hyperuricemia was higher in the RA patients compared to the non-RA subjects (18.1% ± 1.7% at 10 years in RA versus 11.5% ± 1.5% in non-RA; *P* = 0.008, Table 2; Figure 2(a)). However, after randomly resampling RA patient data to mimic the testing rate of non-RA subjects, the difference in cumulative incidence of hyperuricemia between groups disappeared (12.9% ± 1.5% at 10 years in RA versus 11.5% ± 1.5% in non-RA; *P* = 0.64, Table 2; Figure 2(b)). There was no difference in the rate of development of hyperuricemia among RA and non -RA by sex (*P* = 0.61 for RA/non-RA by male/female interaction).

The associations between hyperuricemia and CVD/mortality were more consistent in patients without RA than in patients with RA (Table 3). However, hyperuricemia appeared to be more strongly associated with mortality among the RA (Hazard Ratio [HR]: 2.00; 95% confidence interval [CI]: 1.51, 2.64) than among the non-RA patients (HR: 1.59; 95% CI: 1.12, 2.27), but this difference did not reach statistical significance (interaction *P* = 0.32). In the patients with RA, hyperuricemia was also associated with increased risk of peripheral arterial events (HR: 2.62; 95% CI: 1.27, 5.41; Table 4).

## 4. Discussion

In this population-based study we examined the role of hyperuricemia on cardiovascular disease in patients with and without RA. Among subjects without RA, hyperuricemia was associated with an increased risk of HF and CVD. By contrast, among patients with RA, hyperuricemia was not associated with increased risk of MI, HF, or CVD. However, hyperuricemia was a predictor of peripheral arterial events in RA patients. Hyperuricemia was also a predictor of all-cause mortality in both groups. These findings suggest that in patients with RA hyperuricemia may confer increased risk of peripheral arterial events and all-cause mortality but not necessarily cardiac disease.

Baseline serum uric acid levels in this study were similar to those reported for nationally representative data of adults in the United States [23]. The prevalence of hyperuricemia among healthy US adults has been reported to be 6–8% but increased among persons with hypertension and CVD risk factors [17]. Similarly, in our study, a proportion of subjects had cardiovascular comorbidities and higher rates of hyperuricemia. Baseline prevalence of hyperuricemia was similar between RA patients and non-RA subjects in our study.

RA and gout cooccur infrequently [24]. As a result, data are limited regarding the occurrence of hyperuricemia in patients with RA. We calculated the cumulative incidence of hyperuricemia in patients with and without RA and found that during follow-up, patients with RA appeared to develop hyperuricemia more frequently than their non-RA counterparts. However, when uric acid data for the RA cohort were randomly sampled at the same rate as the non-RA comparison cohort, the cumulative incidence of hyperuricemia in the resampled dataset was similar to that of the non-RA group. This suggests that excess hyperuricemia was incidentally found in RA patients due to higher rates of testing. A small study of healthy subjects found that it is not uncommon to detect transient hyperuricemia with repeated SUA measurements [25].

In many epidemiologic studies of the general population, hyperuricemia has been associated with increased risk of cardiac disease including myocardial infarction [26, 27], HF [11], and coronary heart disease [12]. However, there are also studies which have suggested that uric acid is not an independent risk factor for heart disease [8, 9]. For example, a recent Mendelian randomization study by Palmer et al. used a single nucleotide polymorphism of the SLC2A9 urate transporter gene to evaluate the effects of hyperuricemia on ischemic heart disease and found no association [9]. They suggested that obesity could be an important confounder when studying CVD outcomes. It is still debatable whether uric acid is a causal agent in cardiovascular disease pathogenesis or rather a biomarker of disease.

Subjects in the non-RA cohort of our study were reflective of the general population within Olmsted County. In these patients, hyperuricemia was associated with increased risk of MI, HF, and CVD after adjusting for age, sex, and calendar year. Increased risk for MI did not reach statistical significance, but this was likely due to a small number of observed events. Upon further adjustment for traditional cardiovascular risk factors, hazard ratios for MI and CVD were attenuated while risk of HF increased slightly. These associations were further attenuated after adjusting for reduced kidney function, alcoholism, low-dose aspirin, and urate-lowering therapy.

In contrast, few studies have examined the potential effects of hyperuricemia on CVD in patients with RA. Results from a cross-sectional study of 400 RA patients suggested that higher serum uric acid levels were associated with an increased likelihood of CVD prevalence even after adjusting for confounders (OR 1.36 per 1 mg/dL increase in serum uric acid) [16]. However, in our cohort of RA patients, hyperuricemia was not associated with increased risk of cardiac disease incidence, despite greater numbers of cardiac events compared to non-RA subjects.

Several factors could have affected the observed result. One possibility is that the null hypothesis, that hyperuricemia has no effect on CVD, could be true. However, some patients with transient hyperuricemia were likely included in the analysis due to a higher rate of testing among patients with RA. The inclusion of such patients could dilute the effects of hyperuricemia on cardiovascular outcome. Another consideration is that, on average, patients with RA started the study with lower serum uric acid levels and continued to have lower levels for the first 10 years of disease. Reasons for this are speculative but may relate to medication effects, comorbidity burden. The effect though is that RA patients may have had less exposure to uric acid prior to incidence of hyperuricemia and therefore experience fewer adverse cardiac outcomes related to the exposure. An alternative possibility is that hyperuricemia may have relatively less impact on cardiac processes in the context of disease-specific factors in RA (e.g., systemic inflammation), as has been suggested for some traditional CVD risk factors [7].

In terms of noncardiac vascular outcomes, diseases of the peripheral arteries were examined as a composite outcome that included peripheral arterial disease (PAD), aortic aneurysm, renal artery stenosis, and arterial thromboembolism. Few other studies have looked at the association of uric acid levels with peripheral arterial disease (PAD). One cross-sectional study of the US population found that higher SUA was associated with PAD [28]. A case-control study reported an association between SUA and increased risk of PAD, though the relationship was not statistically significant [29]. Among patients in the RA cohort of our study, hyperuricemia was a predictor for the occurrence of peripheral arterial events even after adjusting for traditional cardiovascular risk factors, including smoking.

Mechanisms which may contribute to vascular disease in patients with hyperuricemia include uric acid's ability to promote endothelial dysfunction [30], decrease nitrous oxide bioavailability, and increase oxidative stress [14]. Uric acid has also been associated with increased inflammatory markers such as C-reactive protein [31] which have been implicated in peripheral vascular disease [32]. Such effects may interact synergistically with the high degree of systemic inflammation in RA to promote increased atherogenesis.

SUA has been found to be associated with all -cause mortality in many but not all studies. A recent meta-analysis found that across studies of the general population, SUA in the highest level (e.g., quartile, quintile) compared to the lowest level was associated with all-cause mortality (pooled RR 1.24; 95% CI: 1.09–1.42) [33]. Interestingly, one study of high-functioning community-dwelling seniors found that inflammation was an important confounder in their analysis of SUA and mortality in women but not in men [34]. Given the high levels of systemic inflammation that affect persons with RA, it is possible that underlying inflammation contributed to the increased risk of all-cause mortality associated with hyperuricemia in the RA cohort of our study.

There were some limitations to our study. Since data were retrospectively collected from the medical record, only clinically obtained data were available. Detailed information about diuretic use was not available. Also, the patient population consisted predominantly of Caucasian patients, so results may or may not necessarily be generalizable to patients of other ethnicities [35]. Strengths of our study include that it was a population-based study with long follow-up duration.

In summary, we evaluated hyperuricemia as a possible risk factor for cardiovascular disease in patients with RA. The results of our study suggest that hyperuricemia may not be a risk factor for excess cardiovascular disease in patients with RA. However, hyperuricemia was associated with increased risk of peripheral arterial events and all-cause mortality. Further research should investigate other nontraditional risk factors of cardiovascular disease in patients with RA.

## Supplementary Material

The Supplementary Material consist of a table containing definitions used for non-cardiac vascular disease.

## Figures and Tables

**Table 1:** Characteristics of 813 patients with rheumatoid arthritis (RA) and 813 subjects without RA (non-RA).

	RA (*n* = 813)	Non-RA (*n* = 813)	*P* value
Age at incidence/index, years, mean ± SD	55.9 ± 15.7	55.9 ± 15.7	0.99
Sex, female, *n* (%)	556 (68%)	556 (68%)	1.0
Race			0.023
White	761 (93.6%)	771 (94.8%)	0.10 (or white versus nonwhite)
American Indian/Alaska native	3 (0.4%)	1 (0.1%)	
Asian	21 (2.6%)	14 (1.7%)	
Black or African American	5 (0.6%)	12 (1.5%)	
Native Hawaiian or other Pacific Islander	4 (0.5%)	0 (0%)	
More than 1 race	11 (1.4%)	3 (0.4%)	
Unknown	8 (1.0%)	12 (1.5%)	
Length of follow-up, years, mean ± SD	9.6 ± 6.9	10.9 ± 7.2	—
Smoking status at incidence/index, *n* (%)			
Never	364 (45%)	435 (54%)	0.002
Current	178 (22%)	144 (18%)	
Former	271 (33%)	234 (29%)	
Body mass index at incidence/index, mean ± SD	27.8 ± 6.2	27.8 ± 7.8	0.74
Diabetes mellitus at incidence/index, *n* (%)	79 (10%)	67 (8%)	0.30
Hypertension at incidence/index, *n* (%)	307 (38%)	275 (34%)	0.10
Dyslipidemia at incidence/index, *n* (%)	444 (55%)	391 (48%)	0.008
eGFR < 60 mL/min/1.73 m^2^ at incidence/index, *n* (%)	74 (9%)	74 (9%)	1.0
Alcoholism at incidence/index, *n* (%)	56 (7%)	55 (7%)	0.92
Low-dose aspirin at incidence/index, *n* (%)	130 (16%)	135 (17%)	0.74
CVD at incidence/index, *n* (%)	95 (12%)	99 (12%)	0.76
Serum uric acid at incidence/index date∗, mg/dL	(*n* = 616)	(*n* = 510)	
Mean ± SD	5.1 ± 2.6	5.2 ± 1.5	0.011
Median, IQR	4.8 (3.7, 5.9)	5.0 (4.1, 6.1)	
Total number of uric acid tests performed∗∗	5231	3258	—
Number of tests per patient, mean (SD)	8.4 (11.7)	6.9 (8.9)	—
Rate of uric acid tests per person-year of follow-up	0.77 per 1 py	0.50 per 1 py	<0.001
Presence of hyperuricemia at incidence/index, *n* (%)	62/616 (10%)	63/510 (12%)	0.22
Use of urate lowering therapy at incidence/index date	7 (1%)	8 (1%)	0.80
Use of urate lowering therapy, ever	28 (3%)	28 (3%)	1.0

*Closest to incidence/index date within ±90 days.

∗∗From incidence/index date to last follow-up.

IQR: interquartile range; *n*: number; SD: standard deviation; py: person-year; eGFR: estimated glomerular filtration rate.

**Table 2:** Cumulative incidence of hyperuricemia in patients with rheumatoid arthritis (RA) compared to subjects without RA (non-RA).

Group	Number of patients at risk (RA/non-RA)	Number of patients with outcome (RA/non-RA)	Cumulative incidence (%) of hyperuricemia (±SE)	*P* value compared to non-RA
Non-RA	502	69	10 yr: 11.5 ± 1.5 20 yr: 16.9 ± 2.0	
RA	630	111	10 yr: 18.1 ± 1.7 20 yr: 24.6 ± 2.3	0.008
RA with testing rate of non-RA∗	630	81	10 yr: 12.9 ± 1.5 20 yr: 18.1 ± 2.0	0.64

*Based on randomly selected uric acid tests for RA patients to mimic lower testing rate of the non-RA cohort.

**Table 3:** Associations between hyperuricemia and cardiovascular disease/mortality in patients with rheumatoid arthritis (RA) and subjects without RA (non-RA).

	*N* events in RA/non-RA	MI HR (95% CI)	CVD HR (95% CI)	HF HR (95% CI)	Death HR (95% CI)
		46/41	137/108	92/69	229/163
Model 1	RA	1.01 (0.50, 2.06)	1.00 (0.66, 1.52)	1.31 (0.82, 2.10)	**2.00** (**1.51, 2.64**)
	Non-RA	1.84 (0.91, 3.72)	**1.81** (**1.14, 2.87**)	**1.88** (**1.09, 3.25**)	**1.59** (**1.12, 2.27**)

Model 2	RA	0.86 (0.41, 1.80)	0.88 (0.57, 1.38)	1.18 (0.71, 1.95)	**1.96** (**1.45, 2.65**)
	Non-RA	1.66 (0.81, 3.40)	1.59 (0.99, 2.55)	**1.95** (**1.13, 3.39**)	**1.57** (**1.09, 2.24**)

Model 3	RA	0.69 (0.31, 1.54)	0.94 (0.58, 1.51)	1.04 (0.61, 1.77)	**1.85** (**1.35, 2.55**)
	Non-RA	1.37 (0.63, 2.96)	1.42 (0.84, 2.39)	1.59 (0.89, 2.84)	**1.51** (**1.03, 2.22**)

Model 1: age, sex, and calendar year adjusted.

Model 2: Model 1, additionally adjusted for smoking, hypertension, obesity (BMI ≥ 30 kg/m^2^), diabetes mellitus, and dyslipidemia.

Model 3: Model 2, additionally adjusted for eGFR < 60, urate lowering therapy, alcoholism, and low-dose aspirin use.

MI: myocardial infarction; CVD: cardiovascular disease (composite of MI, revascularization procedures, angina, and heart failure.); HF: heart failure; Death: all-cause mortality; HR: hazard ratio; CI: confidence interval.

**Table 4:** Associations between hyperuricemia and noncardiac vascular disease in patients with rheumatoid arthritis (RA). Data on subjects without RA were omitted as they were only available for part of the study period (1995–2007).

	*N* events in RA	Cerebrovascular events HR (95% CI)	Peripheral arterial events HR (95% CI)	Venous thromboembolic events HR (95% CI)
		49	36	48
Model 1	RA	1.74 (0.93, 3.23)	**2.62** (**1.27, 5.41**)	1.56 (0.83, 2.95)

Model 2	RA	1.39 (0.72, 2.67)	**2.52** (**1.17, 5.42**)	1.27 (0.66, 2.45)

Model 3	RA	1.23 (0.61, 2.46)	**2.54** (**1.13, 5.70**)	1.44 (0.74, 2.81)

Model 1: age, sex, and calendar year adjusted.

Model 2: Model 1, plus being adjusted for smoking, hypertension, obesity (BMI ≥ 30 kg/m^2^), diabetes mellitus, and dyslipidemia.

Model 3: Model 2, plus being adjusted for eGFR < 60, urate lowering therapy, alcoholism, and low-dose aspirin use.

HR: hazard ratio; CI: confidence interval.
