# Supplementary material for: Hyperuricemia and Incident Cardiovascular Disease and Noncardiac Vascular Events in Patients with Rheumatoid Arthritis
Source: Int J Rheumatol. 2014 Aug 17;2014:523897. doi: 10.1155/2014/523897 (PMC4150464; doi:10.1155/2014/523897)
Supplement: Supplementary file 1 — The Supplementary Material consist of a table containing definitions used for non-cardiac vascular disease. [file 523897.f1.pdf]

Supplementary Table. Definitions used for non-cardiac vascular disease.

1. Hemorrhagic stroke	Clinical diagnosis by neurologist + verified by CT/MRI or autopsy or cerebrospinal fluid analysis
2. Nonhemorrhagic or nonspecified stroke	Clinical diagnosis by neurologist + verified by CT/MRI or autopsy
3. Transient ischemic attack	Clinical diagnosis by neurologist
4. Amaurosis fugax	Clinical diagnosis
5. Aortic aneurysm	Diameter increased >50% compared with normal values; diameter ≥3.0 cm in abdominal aorta; verified by ultrasound/CT or at autopsy
6. Renal artery stenosis	Verified by ultrasound/renal scintigraphy/angiography; assumed clinically significant if detected
7. Peripheral vascular disease or atherosclerosis obliterans	Clinical diagnosis supported by documented vascular physical examination; ankle/brachial index <0.9 or

	angiography confirming disease, if performed
8. Arterial thromboembolism	Clinical diagnosis supported by angiography or autopsy
9. Deep vein thrombosis	Verified by phlebography/venography or ultrasound or autopsy
10. Pulmonary embolism	Verified by angiography, CT angiography, scintigraphy, or autopsy
Cerebrovascular events (1-4 above)	
Peripheral arterial events (5-8 above)	
Venous thromboembolic events (9-10 above)	
